# A hypoxia-inducible factor 1α null splice variant lacking exon 10

**DOI:** 10.1038/cddis.2017.269

**Published:** 2017-06-15

**Authors:** Xiangyu Zhou, Weijia Zeng, Rui Peng, Hongyan Wang

**Affiliations:** 1 Institute of Reproduction & Development, Hospital and Institute of Obstetrics & Gynecology, State Key Laboratory of Genetic Engineering, School of Life Sciences, Collaborative Innovation Centre of Genetics and Development, Fudan University, Shanghai 200011, China

*Dear Editor*,

Hypoxia-inducible factor 1 (HIF1) is a master transcription factor that regulates the expression of hypoxia-inducible genes involved in erythropoiesis, vascular remodeling and glucose metabolism in response to hypoxia. Dysregulation of HIF1α has been heavily implicated in tumor progression.^1^ In this study, using exome sequencing, we identified a synonymous somatic variant of *HIF1α* (c.1257A>G, E419E) in a 40-year-old patient with a primary malignant cardiac tumor. This variant is located in exon 10 of *HIF1α* and near the intron 9/exon 10 boundary. This mutation represents a novel form, and has no frequency record based on human cancer databases, including COSMIC, cBioPortal, as well as in dbSNP and 1000 Genomes. The minor allele frequency in the ExAC database is 3.533e−5 (4/113218). Bioinformatic predictions using SplicePort,^2^ GeneSplicer^3^ and Mutationtaster^4^ uniformly indicated that a new acceptor splice site would be created due to this single base exchange.

To confirm whether alternative splice transcripts are generated, a PCR primer pair was designed to amplify the cDNA fragment from exon 8 to exon 11. Two distinct DNA bands of different sizes were repeatedly observed in cardiac tumor tissue (Figure 1a). However, the lower band was markedly depleted in the tumor-adjacent tissue. Furthermore, we found that this lower band was undetectable in blood sample of patient and in normal heart tissues of spontaneous aborted fetuses (Supplementary Figure S1). Gel extraction and Sanger sequencing revealed that the lower band (373 bp) lacked exon 10 when compared to the upper band (659 bp). In this alternative splice variant, exon 9 and 11 were directly joined, and the altered reading frame was immediately terminated due to two sequential stop codons at presumptive amino acid (aa) positions 418 and 419. This truncated 417-aa variant lacked most of important domains. Thus, we do not expect that this splice variant would exhibit normal transcriptional activity.

Nuclear translocation is fundamental for transcriptional activation. To compare the nuclear location of full-length and the 417-aa HIF1α *in vitro*, subcellular localization and nuclear extraction assays were performed in human HeLa and 293T cells, respectively. Both confocal microscopy and western blot results indicated that the 417-aa HIF1α was mostly retained in the cytoplasm, whereas full-length HIF1α was translocated to the nucleus.(Figure 1b). However, HIF1α 417-aa displayed higher stability in 293T cells due to lack of Pro-564 residue acid, which is essential for proline hydroxylation and VHL-dependent E3 degradation (Supplementary Figure S2). As a consequence, expression of classical HIF1 downstream targets, including VEGFA and VEGFB, as well as PHD2 and PHD3 were downregulated in cardiac tumor tissues. Next, we performed RNA-seq to compare the transcriptomes of full-length and 417-aa HIF1α in 293T cells. Especially, PAK6 (p21 protein activated kinase 6) was upregulated by full-length HIF1α but was markedly downregulated in cells transfected with 417-aa HIF1α (Figure 1c). A previous study found that PAK1 upregulates HIF1α in colorectal cancer.^5^ Whether PAK6 acts as a novel interaction partner of HIF1α requires further investigation.

Different alternative transcripts including HIF1α-827, 785, 736, 557, 516 and 417 have been reported.^6, 7, 8^ however, the roles of these splice variants in human diseases are still unclear. Lee *et al.*^6^ claimed that HIF1α-417, the shortest splice variant, could undergo nuclear translocation and promote transactivation using the transactivation domain of HIF1β however, in our study, both nuclear translocation and transactivation were, at least in part, deficient with this splice variant.

Although our results fail to provide a solid correlation between *HIF1α* (c.1257A>G, E419E) and the premature HIF1α transcript, we found that the 417-aa HIF1α owns higher stability and is deficient with normal nuclear translocation. We postulated that this variant (c.1257A>G) enables HIF1α alternative splicing to compromise excessive HIF1α transcription during the early stages of tumorigenesis.

## Figures and Tables

**Figure 1:**
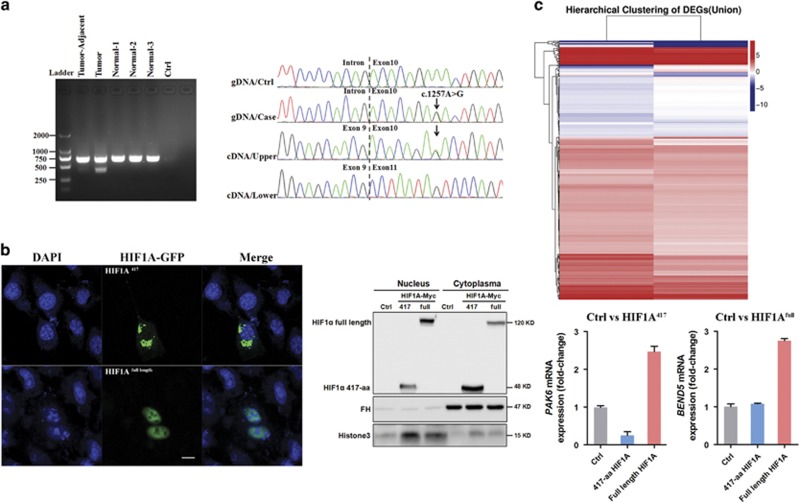
Identification of a *HIF1α* null splice variant in a patient with a cardiac tumor. (**a**) Two distinct bands were clearly observed from cardiac tumor tissue samples (left). Total RNA was extracted from cardiac tumor and tumor-adjacent tissues of patient and normal heart tissues of three aborted fetuses (normal 1-3), then *HIF1α* cDNA was amplified by real-time PCR. *HIF1α* c.1257A>G was confirmed by Sanger sequencing. The lower band (373 bp) represents an alternative splice variant lacking exon 10, when compared to the sequence of the upper band (659 bp) (right). gDNA, genomic DNA. (**b**) Confocal microscopy photos of the 417-aa and full-length HIF1α nuclear localization in HeLa cells (scale bar, 20 μm) (left). Western blots of the 417-aa and full-length HIF1α in nuclear and cytoplasmic extracts from 293T cells (right). FH (fumarase) and Histone 3 served as positive controls for cytoplasmic and nuclear proteins, respectively. (**c**) Hierarchical cluster analysis of DEGs (differentially expressed genes) in Ctrl/HIF1α-417 and Ctrl/HIF1α-full samples. Representative DEGs, including *PAK6* and *BEND5* are validated by qPCR as indicated. Three independent experiments were performed, each sample was repeated in triplicate
