# Supplementary material for: A hypoxia-inducible factor 1α null splice variant lacking exon 10
Source: Cell Death Dis. 2017 Jun 15;8(6):e2873–. doi: 10.1038/cddis.2017.269 (PMC5520924; doi:10.1038/cddis.2017.269)
Supplement: Supplementary Information [file cddis2017269x1.doc]

**Supplementary information**

**1 Methods**

**Subjects**

In this study, a 40-year-old man was diagnosed as having a primary cardiac malignant fibrous histiocytoma (MFH) in the right ventricle by MD Anderson Cancer Center, Houston, USA. Tumor and adjacent tissue samples were taken for biopsy during surgery and then conserved for RNA extraction. In addition, normal heart tissues (right ventricle) from three spontaneous aborted fetuses, peripheral blood samples of the patient and four healthy age-matched control individuals were collected to perform RNA isolation and Real-time PCR. All the procedures in this study were approved by the Medical Ethics Committee of Fudan University and written consent was obtained prior to commencement.

**RNA acceptor splice site prediction**

Online softwares SplicePort (http://spliceport.cbcb.umd.edu/), GeneSplicer (http://ccb.jhu.edu/software/genesplicer/) and Mutationtaster (http://mutationtaster.org/) were utilized to proceed predictions by following instructions. For SplicePort prediction, we use HIF1A intron-exon10 acceptor AG splice site neighborhood of 80 nucleotides upstream and 80 nucleotides downstream. The threshold of -0.5 produces a Sensitivity value of 96.65% and a False Positive Rate of 4.34% for AG location. For GeneSplicer prediction, we use HIF1A intron-exon10 acceptor AG splice site neighborhood of 30 up- and downstream nucleotides. For acceptor splice site prediction, the Sensitivity (%) was chosen at 95% and the maximum score within bp is 60.

**Real time and quantitative PCR**

The alternative splice transcript of HIF1ɑ was studied via real-time PCR. cDNA was obtained from the reverse transcription of 1000 ng total RNA from tumor and adjacent normal tissue of patient-1 as well as blood samples of 4 control persons by using the QuantScript RT Kit (Tiangen). Amplification of HIF1A was performed using 20 ng/µl of template cDNA with primers (Forward:5'-ACAAGTCACCACAGGACAGTAC-3',Reverse:5'-ATTGACCATATCACTATCCACA -3'). Expression level was directly visualized by running DNA agarose gel.

In the quantitative RT-PCR , the 417-aa HIF1A was distinguished from full length HIF1A by using following primers: Forward:5’-AGATTTTGGCAGCAACGCCTA-3’, Reverse:5’-CATCTCCAA GTCTAAATCTGTG-3’. The last four bases (CCTA) of forward primers were from exon 11 and exon 10 was skipped. Then SuperReal SYBR Geen PreMix (TIANGEN) was prepared to perform standard quantitative real-time PCR reactions by using ABI StepOne. All quantitative RT-PCR reactions were run in triplicate. In addition, differential expressed genes PAK6 and BEND5 (RNA-seq data) were validated by qPCR following aforementioned procedures. mRNA levels of VEGFA, VEGFB, PHDs, HIF-2ɑ were analyzed from transcriptome data based on tumor/tumor-adjacent tissues.

**Plasmids Construction**

The *HIF1ɑ (*[NM_001530](https://www.ncbi.nlm.nih.gov/nuccore/NM_001530.3)) expression vector (pENTER-C-Flag) was purchased from Vigenebio (Maryland, USA). Full length 826-amino acid and truncated 417-amino acid HIF1ɑ splice transcripts were amplified and cloned into pCMV6-C-GFP or pCMV6-C-Myc vector by using Sgf1 and MluI restriction enzyme sites.

**Subcellular Localization**

Hela cells were plated in 6-well plates and transiently transfected with GFP-tagged full-length and 417-aa *HIF1ɑ* to overexpress recommbinant protein using Lipofectamine 3000 (Thermo Fisher). After 36 hrs, cells were fixed in 4% PFA (paraformaldehyde) for 10 min and washed in PBS three times. Permeabilize cells in 0.1% Triton-X-100 for 10 minutes, followed by three more PBS washes. Cells were stained in 500 ul/well of DAPI (1:2000 dilution in 1xPBS) for 8 min at room temperature and then photographed with a laser scanning confocal microscope (LSM710;Leica). The DAPI and GFP were excited at 350 nm and 488 nm wavelength, respectively.

**Immunoblot Analysis**

293T cells were transfected with the Myc-tagged 417-aa and full length HIF1ɑ. After 24 hrs, cells were collected to perform Nuclear and Cytoplasmic Extraction assay by following standard protocol (Thermo Scientific) or were homogenized in RIPA Buffer (Sangon Biotech) supplemented with a 1:200 dilution of protein inhibitor (Sangon Biotech). Following electrophoresis and membrane transfer, proteins were probed with one of the following primary antibodies in either 5% non-fat milk or 5% BSA: anti-Myc (#14038), anti-actin (#4970), anti-Hydroxy-HIF1ɑ (Pro564)(#3434), anti-FH (#4567) and anti-Histone H3(9717) were from CST(Cell Signaling Technology). After incubation with secondary HRP-antibodies (either anti-rabbit [7074; CST] or anti-mouse [8270; Sigma-Aldrich]), chemiluminescent detection was performed using ECL1/2 (PC198506/1859701; ThermoFisher).

**RNA-SEQ**

RNA sequencing was performed at BGI Co.,Ltd by using BGISEQ-500 system. Briefly, 293T were transfected with Myc-tagged full-length and 417-amino acid *HIF1ɑ* to overexpress recommbinant protein using Lipofectamine 3000 (Thermo Fisher). After 36 hrs, cells were collected to perform total RNA isolation. The cDNA library was prepared with 1 ug total RNA for each sample and sequencing was performed according to standard manufacture’s protocol from BGI in the previous study [1,2]. The average clean reads number of three samples was 25.02 million and average genome mapping ratio is 90.86%. Gene expression level was calculated by RPKM (Reads Per Kilobase of exon model per Million mapped reads) according to the provided standard formula. Genes with expression fold-change <2 were excluded from subsequent analyses.

**2 Supplementary Figure Legends**

**Figure S1**. The 417-aa HIF1ɑ was detected in cardiac tumor tissues. (a) Quantitative Real-time PCR were performed to determine the expression of the 417-aa HIF1ɑ in cardiac tumor tissue and blood samples of patient. (b) HIF1ɑ was amplified from cDNA generated from blood samples of patient and four health individuals (Ctrl 1-4). mRNA expression levels were directly visualized by running DNA agarose gel.

**Figure S2.** The 417-aa HIF1ɑ possesses higher stability than the full length HIF1ɑ at 48 hr post-transfection (a) due to lack of Pro-564 residue acid which prevents hydroxylation by PHDs on Pro-564(b). 293T cells were transfected with the Myc-tagged 417-aa and full length HIF1a, then cells were collected at 24 hr and 48 hrs post-transfection, respectively, to proceed Western blot. β-actin served as a loading control. The band at 48kd indicates the 417-aa HIF1a and the band at 120kd indicates the full length HIF1a.

**References**

1. Fehlmann T, Reinheimer S, Geng C, et al. cPAS-based sequencing on the BGISEQ-500 to explore small non-coding RNAs[J]. Clinical Epigenetics, 2016, 8(1): 123.
2. Zhang B, Zhang W, Nie R E, et al. Comparative transcriptome analysis of chemosensory genes in two sister leaf beetles provides insights into chemosensory speciation[J]. Insect Biochemistry and Molecular Biology, 2016, 79: 108-118.
