# Supplementary figures and images for: A hypoxia-inducible factor 1α null splice variant lacking exon 10
Source: Cell Death Dis. 2017 Jun 15;8(6):e2873–. doi: 10.1038/cddis.2017.269 (PMC5520924; doi:10.1038/cddis.2017.269)

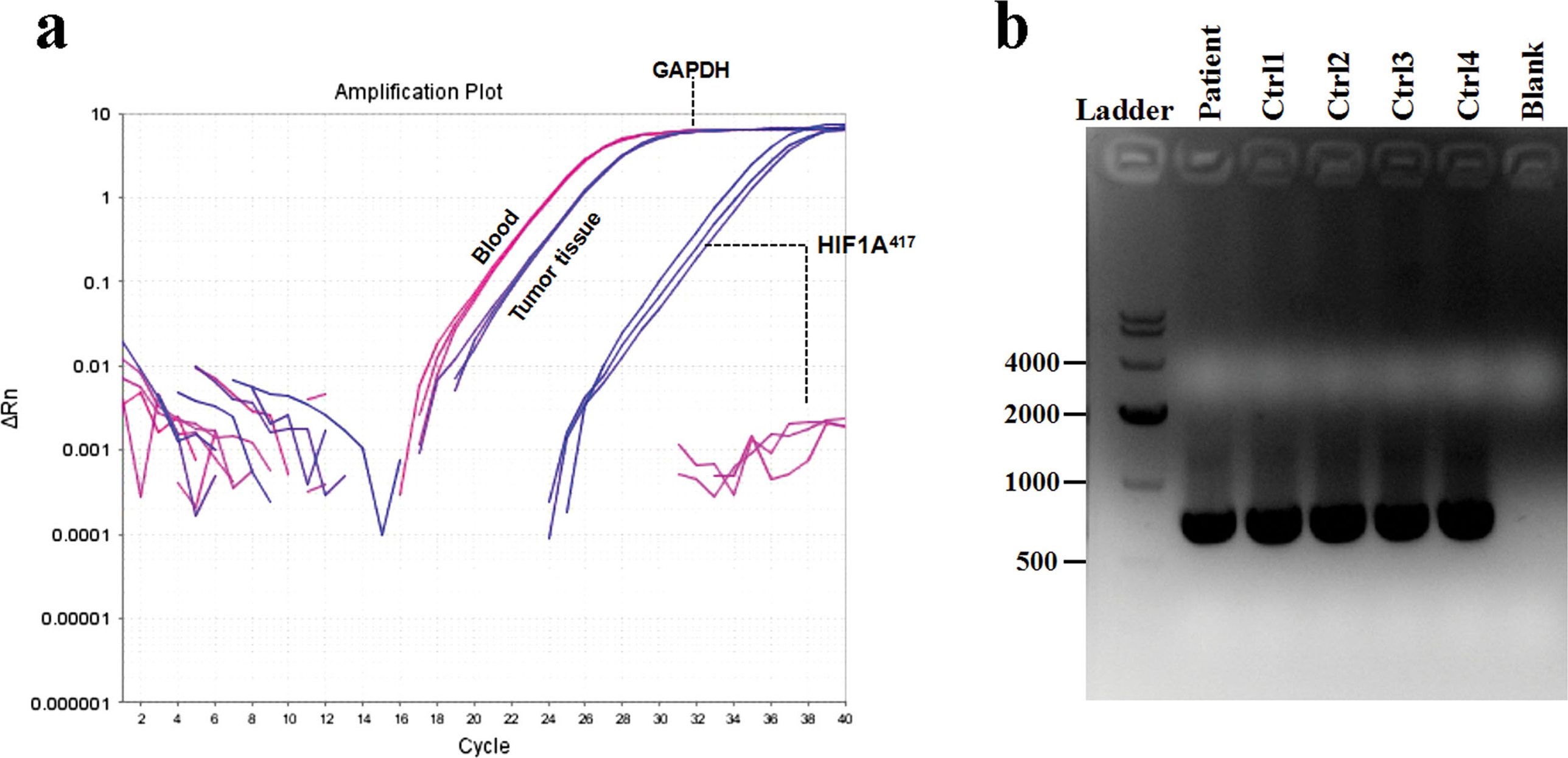

Supplement: Supplementary Figure S1 [file cddis2017269x2.tif]

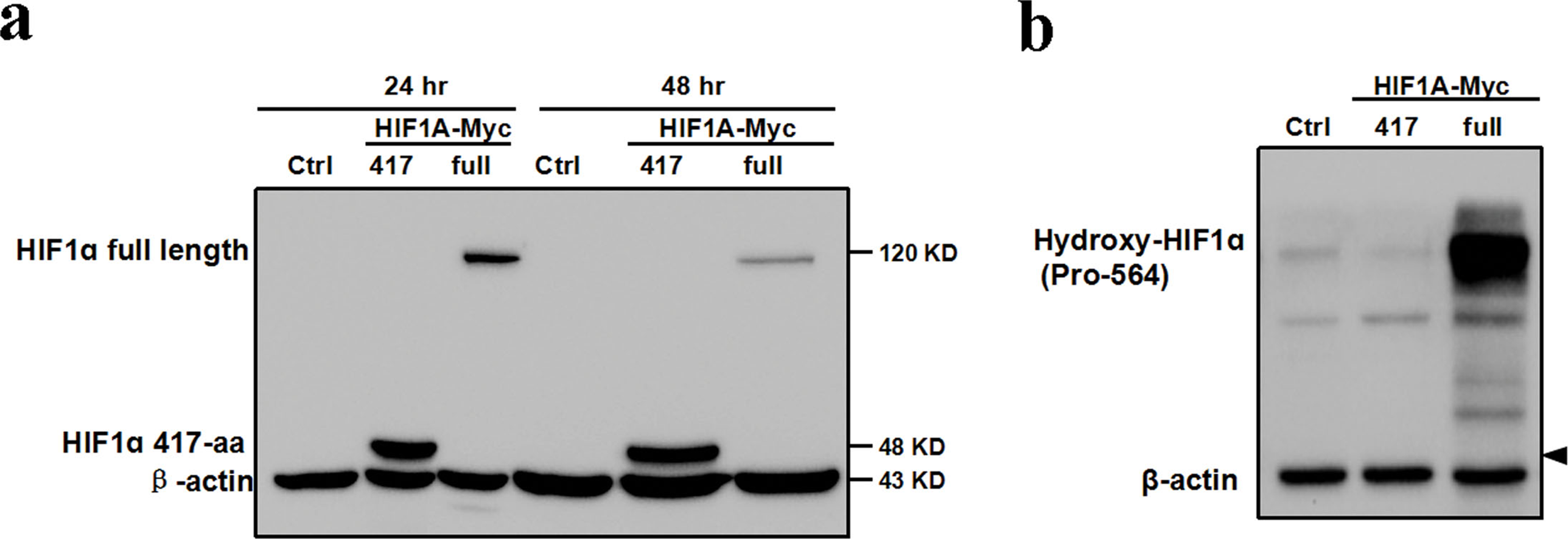

Supplement: Supplementary Figure S2 [file cddis2017269x3.tif]
